# High-throughput proteomic analysis reveals systemic dysregulation in virally suppressed people living with HIV

**DOI:** 10.1172/jci.insight.166166

**Published:** 2023-06-08

**Authors:** Nadira Vadaq, Yue Zhang, Wilhelm A.J.W. Vos, Albert L. Groenendijk, Martinus J.T. Blaauw, Louise E. van Eekeren, Maartje Jacobs-Cleophas, Lisa van de Wijer, Jéssica Cristina dos Santos, Muhammad Hussein Gasem, Leo A.B. Joosten, Mihai G. Netea, Quirijn de Mast, Jingyuan Fu, André J.A.M. van der Ven, Vasiliki Matzaraki

**Affiliations:** 1 Department of Internal Medicine, Radboudumc Center for Infectious Diseases, Radboud Institute of Health Science, Radboud University Medical Center, Nijmegen, Netherlands.; 2 Center for Tropical and Infectious Diseases, Faculty of Medicine, Diponegoro University, Dr. Kariadi Hospital, Semarang, Indonesia.; 3 Department of Genetics, University of Groningen, University Medical Center Groningen, Groningen, Netherlands.; 4 Department of Internal Medicine, OLVG, Amsterdam, Netherlands.; 5 Department of Medical Microbiology and Infectious Diseases, Erasmus Medical Center, Rotterdam, Netherlands.; 6 Department of Internal Medicine, Elizabeth-Tweesteden Ziekenhuis, Tilburg, Netherlands.; 7 Department of Medical Genetics, Iuliu Hatieganu University of Medicine and Pharmacy, Cluj-Napoca, Romania.; 8 Department of Immunology and Metabolism, Life and Medical Sciences Institute, University of Bonn, Bonn, Germany.; 9 Department of Pediatrics, University of Groningen, University Medical Center Groningen, Groningen, Netherlands.

**Keywords:** AIDS/HIV, Bioinformatics, Cardiovascular disease, Innate immunity

## Abstract

**BACKGROUND:**

People living with HIV (PLHIV) receiving antiretroviral therapy (ART) exhibit persistent immune dysregulation and microbial dysbiosis, leading to development of cardiovascular diseases (CVDs). We initially compared plasma proteomic profiles between 205 PLHIV and 120 healthy control participants (HCs) and validated the results in an independent cohort of 639 PLHIV and 99 HCs. Differentially expressed proteins (DEPs) were then associated to microbiome data. Finally, we assessed which proteins were linked with CVD development in PLHIV.

**METHODS:**

Proximity extension assay technology was used to measure 1,472 plasma proteins. Markers of systemic inflammation (C-reactive protein, D-dimer, IL-6, soluble CD14, and soluble CD163) and microbial translocation (IFABP) were measured by ELISA, and gut bacterial species were identified using shotgun metagenomic sequencing. Baseline CVD data were available for all PLHIV, and 205 PLHIV were recorded for development of CVD during a 5-year follow-up.

**RESULTS:**

PLHIV receiving ART had systemic dysregulation of protein concentrations, compared with HCs. Most of the DEPs originated from the intestine and lymphoid tissues and were enriched in immune- and lipid metabolism–related pathways. DEPs originating from the intestine were associated with specific gut bacterial species. Finally, we identified upregulated proteins in PLHIV (GDF15, PLAUR, RELT, NEFL, COL6A3, and EDA2R), unlike most markers of systemic inflammation, associated with the presence and risk of developing CVD during 5-year follow-up.

**CONCLUSION:**

Our findings suggest a systemic dysregulation of protein concentrations in PLHIV; some proteins were associated with CVD development. Most DEPs originated from the gut and were related to specific gut bacterial species.

**TRIAL REGISTRATION:**

ClinicalTrials.gov NCT03994835.

**FUNDING:**

AIDS-fonds (P-29001), ViiV healthcare grant (A18-1052), Spinoza Prize (NWO SPI94-212), European Research Council (ERC) Advanced grant (grant 833247), and Indonesia Endowment Fund for Education.

## Graphical abstract

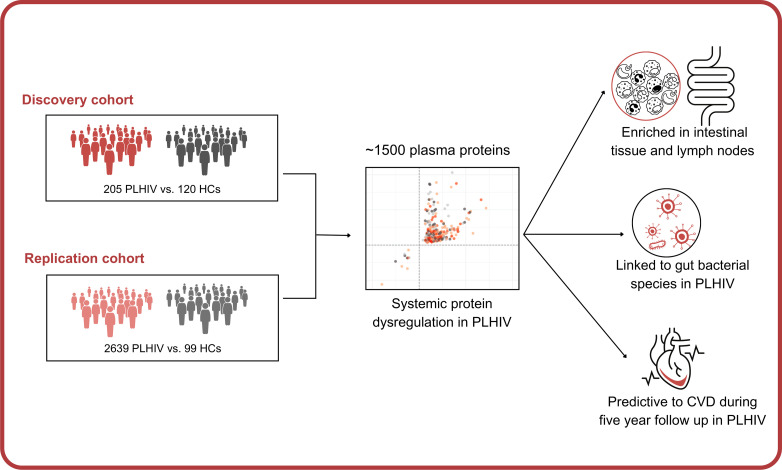

## Introduction

Despite the improved survival rate of people living with HIV (PLHIV) because of efficient virus suppression after the use of antiretroviral therapy (ART), PLHIV are still burdened by excess risk of morbidities and mortality compared with uninfected individuals (1–3). A substantial reduction in deaths related to AIDS is currently leading to an aging HIV-infected population, subsequently resulting in an increase in the proportion of PLHIV with age-related, non-AIDS comorbidities, such as cardiovascular diseases (CVDs) (1, 4–6). These non-AIDS comorbidities are currently the leading cause of death among PLHIV in high-income countries (7–9). Apart from the traditional risk factors (namely, smoking, hypertension, diabetes, and hyperlipidemia), HIV-associated chronic low-grade inflammation, persistent immune dysregulation, and microbial dysbiosis were also recognized as major contributing factors to the development of CVD in PLHIV (10–12). However, the underlying molecular mechanisms and biological pathways mediating chronic immune dysregulation remain unclear, hindering the discovery and development of novel treatment strategies to mitigate the risk of CVD in PLHIV treated with long-term ART.

High-throughput omics technologies can advance our understanding of HIV pathobiology because they enable a thorough and unbiased investigation of the underlying molecular processes in the HIV population. In particular, technologies for proteomic profiling can facilitate the monitoring of numerous cellular processes through the detection of a wide range of proteins, including intracellular, secreted, and low-abundance proteins. Previous proteomic studies in PLHIV receiving ART provided substantial insight into the pattern of inflammation in chronic HIV infection in relation to non-AIDS comorbidities (13–21). However, all previous studies, to our knowledge, focused on a limited number of proteins, and a systematic study of proteins is still lacking.

By assessing 92 inflammatory proteins in the circulation, we previously identified dysregulation of specific pathways in PLHIV and identified 2 inflammatory clusters within the PLHIV that relate to the risk of developing malignancy and CVD (22). In the present study, our aim was to compare the abundance of a wide range of proteins (*n* = 1,472 proteins) that mirror inflammatory, oncology, neurology, and cardiovascular processes in virally suppressed PLHIV and healthy control individuals (HCs). We used the plasma samples of 2 independent cohorts of PLHIV treated with long-term ART and HCs, which are part of the Human Functional Genomics Project (HFGP) (23). Given that persistent immune dysregulation and microbial dysbiosis are major contributing factors to the development of long-term comorbidities in the PLHIV (10, 11), we also examined the relationship of the measured plasma proteins with the bacterial species and the development of CVD in PLHIV.

## Results

### Systemic dysregulation of circulating protein concentrations in PLHIV.

**Figure 1:**
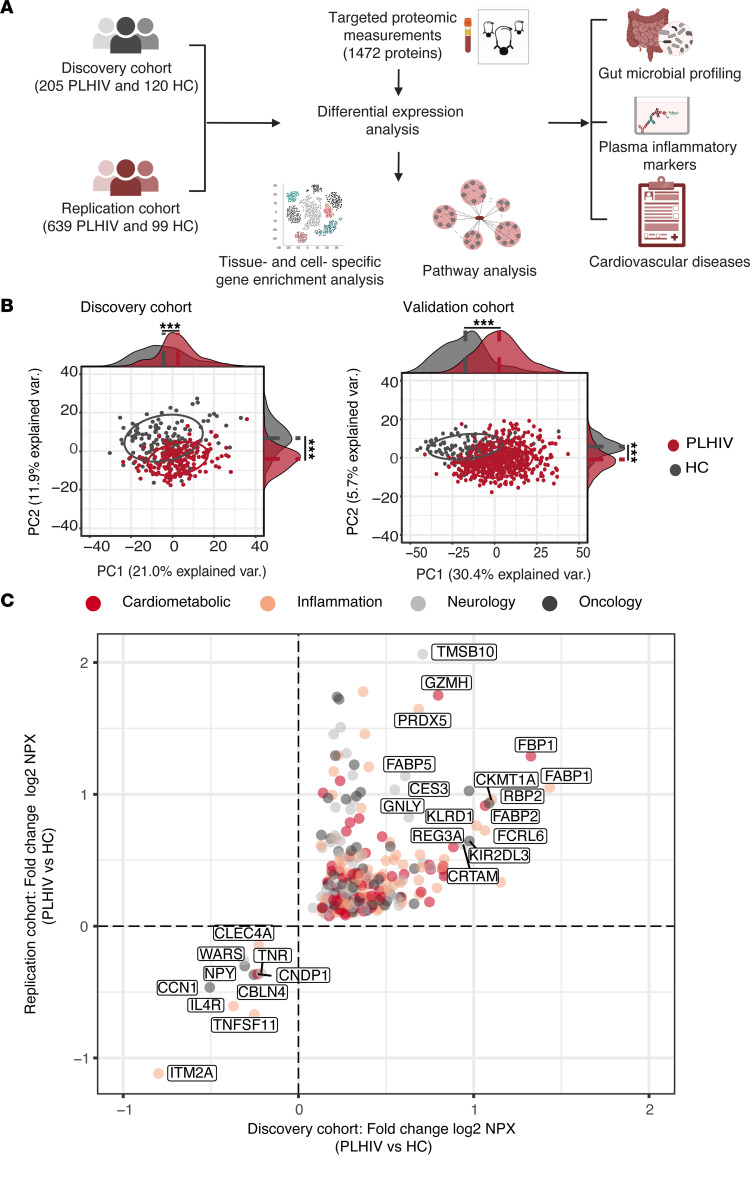
Dysregulation of protein concentrations in PLHIV compared with HCs. (**A**) Study overview. (**B**) PCA of protein levels from the discovery (left: *n* = 205 PLHIV vs. 120 HCs) and replication cohorts (right: *n* = 639 PLHIV vs. 99 HCs) using the first 2 principal components. The ellipses were centered on the basis of the median of PC1 and PC2 for each group (PLHIV and HCs) and the median differences between groups were assessed by the Mann-Whitney *U* test. ***P < 0.0001. (**C**) Four-quadrant plot of the fold-change of sDEPs (*n* = 276) in the discovery (*x* axis; *n* = 205 PLHIV vs. 102 HCs) and replication cohorts (*y* axis; *n* = 639 PLHIV vs. 84 HCs). DE analysis was performed using a linear regression model with age, sex, and smoking status as covariates. Fold-change in the *x* axis label refers to the difference in the mean of log_2_ NPX values between PLHIV and HCs. Only proteins with FDR < 0.05 and log_2_ fold-change ≥ 1.5 are annotated. See also Supplemental Table 4. Var, variation.

The present study had a 2-phase design and included discovery and replication cohorts of PLHIV and HCs from the HFGP. The study overview is depicted in Figure 1A, and the baseline characteristics of study participants are listed in Table 1. The discovery cohort consisted of 205 PLHIV and 120 HCs. PLHIV in the discovery cohort were significantly older (PLHIV vs. HCs: median age [IQR]), 52.4 (13.2) vs. 36.2 (29.4) years, respectively), predominantly consisted of men (*n* = 189 of 205 [92%] vs. *n* = 77 of 120 [64%], respectively), and more often smoked compared with the HCs (*n* = 62 of 205 [30%] vs. *n* = 14 of 103 [14%], respectively) (Table 1). The independent replication cohort consisted of 639 PLHIV and 99 HCs. PLHIV from the replication phase showed similar baseline characteristics as in the discovery phase (Table 1). They were slightly older (median [IQR], 53 [IQR 20.8] vs. 48.6 [IQR 15] years, respectively), predominantly were men (*n* = 585 of 639 [92%] vs. *n* = 75 of 99 [76%], respectively), and more often smoked (*n* = 184 of 639 [29%] and 10 of 87 [12%], respectively) than HCs (Table 1).

First, principal component analysis (PCA) of the protein expression data showed limited overlap between PLHIV and HCs in both the discovery and replication cohorts (Figure 1B), indicating a unique proteomic profile in PLHIV compared with HCs. Next, for 1,309 of 1,463 proteins that passed quality control (QC) in the discovery cohort, we tested which proteins are differentially expressed between the PLHIV and HCs (discovery phase). After adjustment for age, sex, and smoking status, we identified 773 proteins (59%) that were differentially expressed (FDR < 0.05) (Supplemental Table 2; supplemental material available online with this article; https://doi.org/10.1172/jci.insight.166166DS1). Among 773 differentially expressed proteins (DEPs) in the discovery cohort, 403 (52%) were significantly differentially expressed in PLHIV compared with HCs in the replication cohort (*P* < 0.05, adjusted for age, sex, and smoking status) (Supplemental Table 3). We identified 276 significantly DEPs with consistent direction between the 2 cohorts (FDR < 0.05 for the discovery cohort and *P* < 0.05 in the replication cohort; Figure 1C). A list of the shared DEPs (sDEPs) can be found in Supplemental Table 4. The sDEPs were equally distributed across their respective Olink proteomic panels (i.e., cardiometabolic, inflammation, neurology, and oncology) (Supplemental Figure 2), suggesting a multisystem perturbation of plasma protein concentrations in well-treated PLHIV.

Most of the sDEPs were upregulated (*n* = 266 of 276) in PLHIV, with only 10 proteins being downregulated. Next, we ordered the sDEPs on the basis of fold-change and identified 16 sDEPs with fold-change of at least 1.5 in both discovery and replication cohorts (Figure 1C). Of note, these proteins are involved in the uptake, transport, and metabolism of lipids. For example, members of the cytosolic fatty acid–binding protein (FABPs) family (FABP1, FABP2, FABP5, and RBP2) and CES had the highest fold-change among sDEPs. FABP2 (also known as IFABP, which is the term we will use throughout) and REG3A are highly expressed in the intestinal epithelium and have been identified as gut damage markers in PLHIV (24, 25). Other sDEPs with fold-change of at least 1.5 include proteins that are involved in NK cell biology and function (GZMH, KLRD1, FCRL6, GNLY, KIR2DL3), T cell–associated molecules (CRTAM, GLNY, KIR2DL3), gluconeogenesis regulatory enzyme (FBP1), mitochondrial creatine kinase (CKMT1A, CKMT1B), antioxidant enzymes (PRDX5), and proteins with an oncogenic or tumor-suppressive role (TMSB10). Furthermore, 10 proteins that were downregulated include proteins that are highly expressed in the brain and associated with behavioral or psychiatric disorders (NPY, CNDP1, CBLN4, TNR), promote gut mucosal healing (CCN1), are related to inflammatory responses (CLEC4A, IL4R, TNFSF11), and are involved in bone resorption and remodeling (ITM2A, TNFSF11).

### Pathway-, tissue-, and cell-specific enrichment analysis of sDEPs pinpoint persistent intestinal damage in PLHIV.

**Figure 2:**
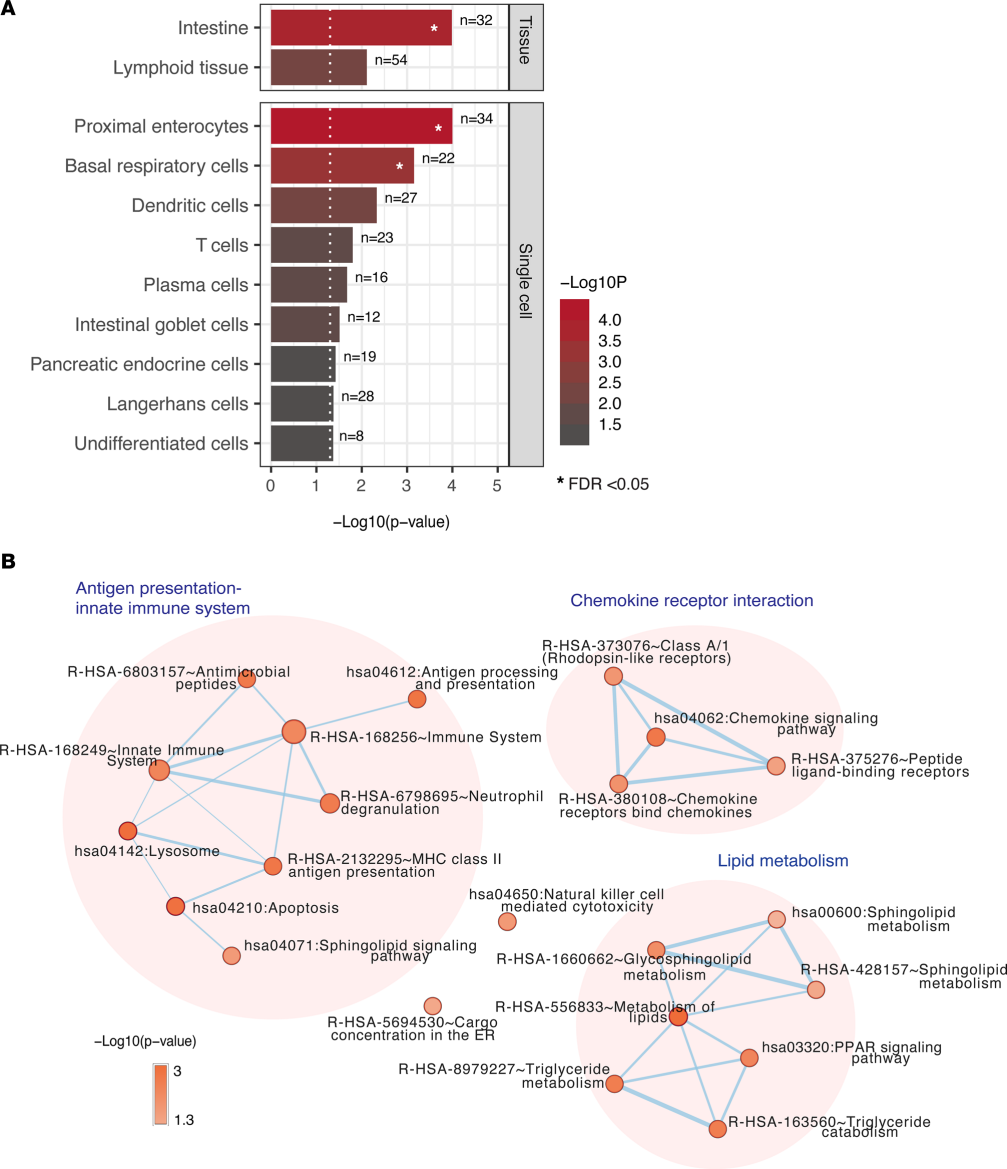
Origin and functional characterization of DEPs between PLHIV and HCs. (**A**) Bar chart showing significantly enriched tissue- and cell-specific proteins using the upregulated sDEPs from the discovery and replication cohorts. Enrichment analysis was performed using a tissue- and a cell-specific gene list according to the consensus transcriptomic data from the HPA and GTEx. See also Supplemental Tables 5 and 6. (**B**) Network of functional pathways based on enrichment analysis of sDEPs. Circular nodes represent pathways, with the colors showing a gradient of the enrichment *P* values; node size represents the number of genes in the pathway; weighted edges represent the degree of gene overlap score between pathways, calculated by the average between the jacquard and overlap coefficients. Clusters of nodes were identified by Markov cluster algorithm (see Methods). See also Supplemental Table 8.

To explore the tissue and cellular origin of upregulated sDEPs (*n* = 266), we performed tissue- and cell-specific enrichment analysis of the genes encoding for the sDEPs using tissue- and cell-specific transcriptomic data from the Human Protein Atlas (HPA) (26) and Genotype-Tissue Expression (GTEx) (27). At the tissue level, the genes coding for the sDEPs were significantly enriched in the intestine- and lymphoid tissue–specific genes (Figure 2A and Supplemental Table 5). Furthermore, at the single-cell level, we found significant enrichment of glandular epithelial cell–specific genes in sDEPs genes, such as proximal enterocytes, basal respiratory cells, and intestinal goblet cells, followed by immune cells (dendritic cells, T cells, plasma cells, and Langerhans cells) and endocrine cells (pancreatic endocrine cells) (Figure 2A and Supplemental Table 6). The 2 most enriched cell groups, enterocytes and intestinal goblet cells, are present in the gastrointestinal tract (GIT), supporting its physiological function and role as a barrier against pathogens in the GIT, especially in the intestine.

In light of the enrichment of sDEPs in genes specific to immune cells, we aimed to explore potential associations between sDEPs and the proportion of innate and adaptive circulating immune cells measured by flow cytometry in PLHIV in the discovery cohort. In general, our analysis revealed positive associations between sDEPs (*n* = 276) and the proportion of circulating immune cells (Supplemental Figure 3 and Supplemental Table 7). Consistent with the tissue and cell type enrichment analyses, the sDEPs were particularly associated with an increase in the proportion of a variety of adaptive immune cells (CD8^+^ T cells, CD8^+^ naive T cells, CD8^+^ effector T cells, B cells, CD4^+^ naive T cells, Th2 cells, Th17 cells, and Tregs) and innate immune cells (neutrophils). Notably, we found that higher relative concentrations of sDEPs were associated with an imbalance between the Th17 cell to Treg ratio, favoring the latter. Th17 cells play a crucial role in maintaining gut mucosal immunity and primarily reside at barrier surfaces in the GIT (28). The loss of Th17 to Treg ratio balance has been linked to disease progression during HIV and SIV infections and may contribute to dysregulation of mucosal immunity, microbial translocation, and chronic immune activation in PLHIV (29–31).

Furthermore, to examine the effects of dysregulation of plasma protein concentration in PLHIV on biological pathways, we performed a pathway enrichment analysis using the 276 sDEPs and visualized the results in the network-based framework (Figure 2B). In general, we identified 3 clusters of pathways related to (a) antigen presentation and the innate immune system, (b) cytokine and chemokine receptor interaction, and (c) the lipid metabolism pathway (Figure 2B and Supplemental Table 8). Among the top most enriched pathways were the following: metabolism of lipids, lysosome, apoptosis, antigen processing and presentation, and MHC class II antigen presentation.

We next tested whether the sDEPs from the intestine and lymphoid tissue were enriched in sDEPs belonging to the immune- or lipid-related pathways (Supplemental Figure 4). The proportion of lymph node–specific proteins was significantly higher in the immune-related pathways than expected (*P* < 0.001). A higher proportion of intestinal proteins was identified in the lipid-related pathways compared with nonintestinal proteins (19% vs. 11%, respectively).

Considering that most of the identified sDEPs were specific to the intestine and lymphoid tissues and have a known role in the immune system, we correlated sDEPs (*n* = 276) with established markers of inflammation that relate to non-AIDS comorbidities (soluble CD163 [sCD163], sCD14, IL6, high-sensitivity C-reactive protein [hsCRP], and D-dimer) (32–34) and microbial translocation (IFABP) (24) measured in PLHIV in the discovery cohort. In general, sDEPs were positively associated with markers of inflammation (FDR < 0.05, linear regression model adjusted by age, sex, and smoking status). As expected, higher relative concentrations of intestine-specific sDEPs, including IFABP, were mainly associated with higher absolute concentrations of IFABP (Supplemental Figure 5).

### Minimal association between sDEPs and HIV-related factors.

To explore whether HIV-related factors may be involved in the dysregulation of sDEPs in PLHIV, associations between sDEP and immunologic and virologic measures of HIV (CD4 nadir and latest, CD4 to CD8 ratio, and HIV RNA zenith and latest) as well as HIV and ART duration were analyzed using 2 independent cohorts of PLHIV (Supplemental Figure 6). Of 276 sDEPs, 8 and 67 were significantly correlated with at least 1 of the HIV-related parameters in the PLHIV of the discovery and replication cohorts, respectively (FDR < 0.05, partial Spearman’s correlation adjusted for age, sex, and smoking status). Of note, PLHIV with a lower CD4 to CD8 ratio had higher relative expression of NK cell antigen KLRD1, CD8A, and cytotoxic genes (GZMA and GZMH) in 2 independent HIV cohorts (Supplemental Figure 6).

### Bacterial species were associated with intestine- and lymph node–specific sDEPs.

**Figure 3:**
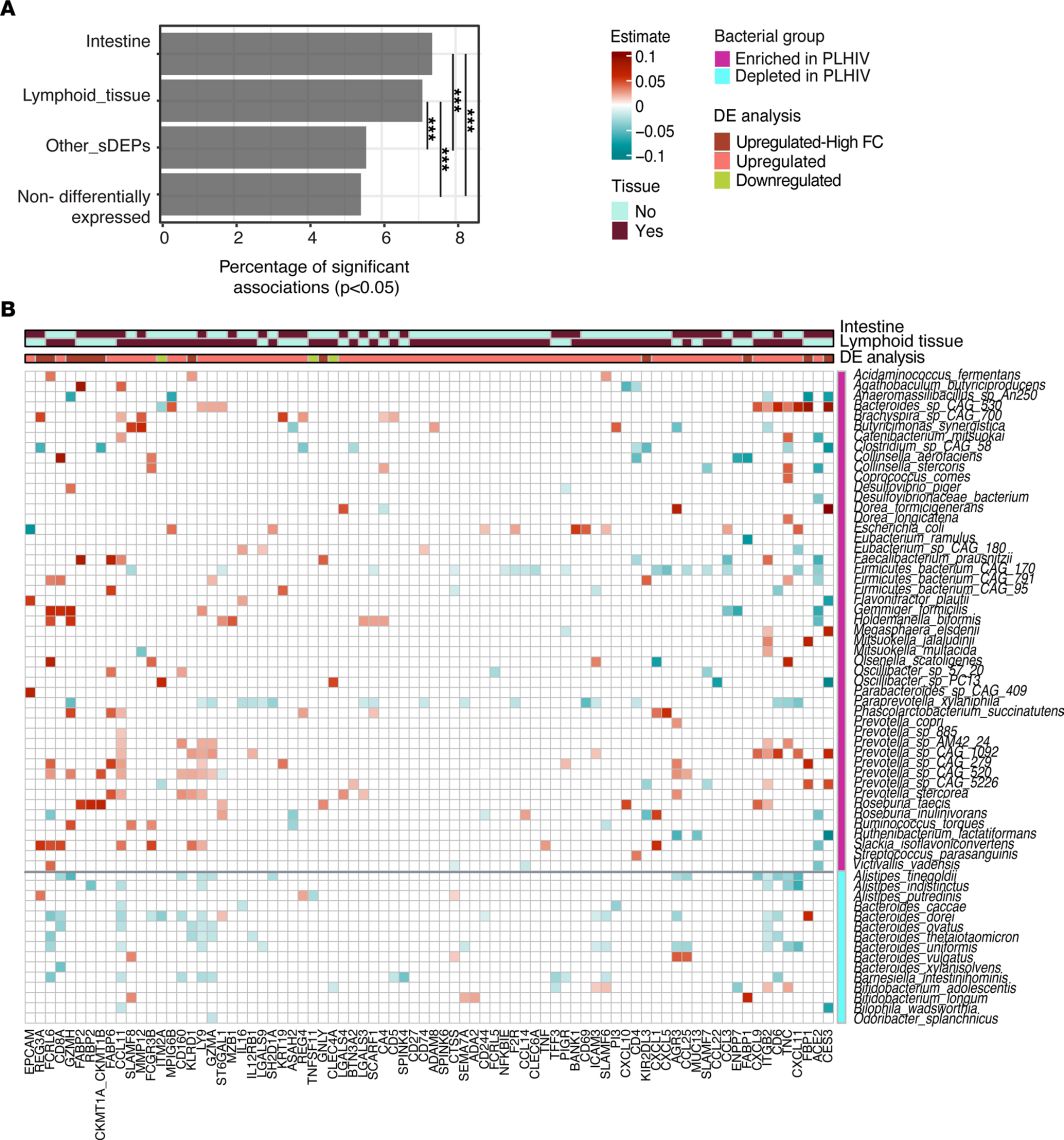
Gut bacterial species were related to sDEPs originated from the intestine and lymphoid tissues in PLHIV. (**A**) Bar chart showing the proportion of significant associations (*P* < 0.05) among 4 different groups of proteins and bacterial species using linear regression analysis corrected for age, sex, smoking status, and read counts. Differences in the proportion of significant associations between bacterial species and protein groups were tested using post hoc pairwise χ^2^ tests. ***P < 0.0001. (**B**) Heatmap depicting the associations between sDEPs specific to the intestine and lymphoid tissues with the abundance of enriched and depleted bacterial species in PLHIV of the discovery cohort (*n* = 143). The analysis was performed using linear regression analysis corrected for age, sex, smoking status, and read counts. Only significant associations (*P* < 0.05) are shown. See also Supplemental Table 9.

Given that both intestine- and lymphoid tissue–specific genes were significantly enriched in our sDEPs, we explored whether gut bacterial composition in PLHIV was linked to the upregulation of plasma protein concentrations. We first investigated the associations between bacterial species and all measured proteins (*n* = 1,472). Among the top most significant associations were proteins that are highly expressed in the intestine and/or lymphoid tissue, such as CCL11 and ACE2 (*P* < 0.0001) (Supplemental Table 9). Next, we compared the proportion of significant associations between bacterial species and proteins (*P* < 0.05, linear regression model adjusted for age, sex, smoking status, and read counts) across the following groups: (i) intestine-specific sDEPs, (ii) lymphoid tissue–specific sDEPs, (iii) sDEPs not specific to the intestine and lymphoid tissues, and (iv) proteins that were not differentially expressed between PLHIV and HCs. We observed a higher proportion of significant associations between bacterial species and sDEPs specific to the intestine and lymphoid tissue compared with the 2 other groups (*P* < 0.001 for all, post hoc pairwise χ^2^ tests; Figure 3A).

Furthermore, we explored whether sDEPs specific to the intestine and lymph node were associated with the abundance of bacterial species that we previously found to be enriched or depleted in PLHIV (35). We found that enriched and depleted bacterial species in PLHIV were positively and negatively associated with intestinal (*n* = 32) and lymph node–specific sDEPs (*n* = 57), respectively (*P* < 0.05, linear regression model adjusted for age, sex, smoking status, and read counts; Figure 3B and Supplemental Table 9). None of the associations remained significant after correcting for multiple testing. Some of the proteins have been linked to gut bacterial composition in previous studies of healthy populations, including EPCAM, TFF3, and REG3A (36).

### Upregulated DEPs were linked to baseline CVDs and risk of developing CVDs in PLHIV.

**Figure 4:**
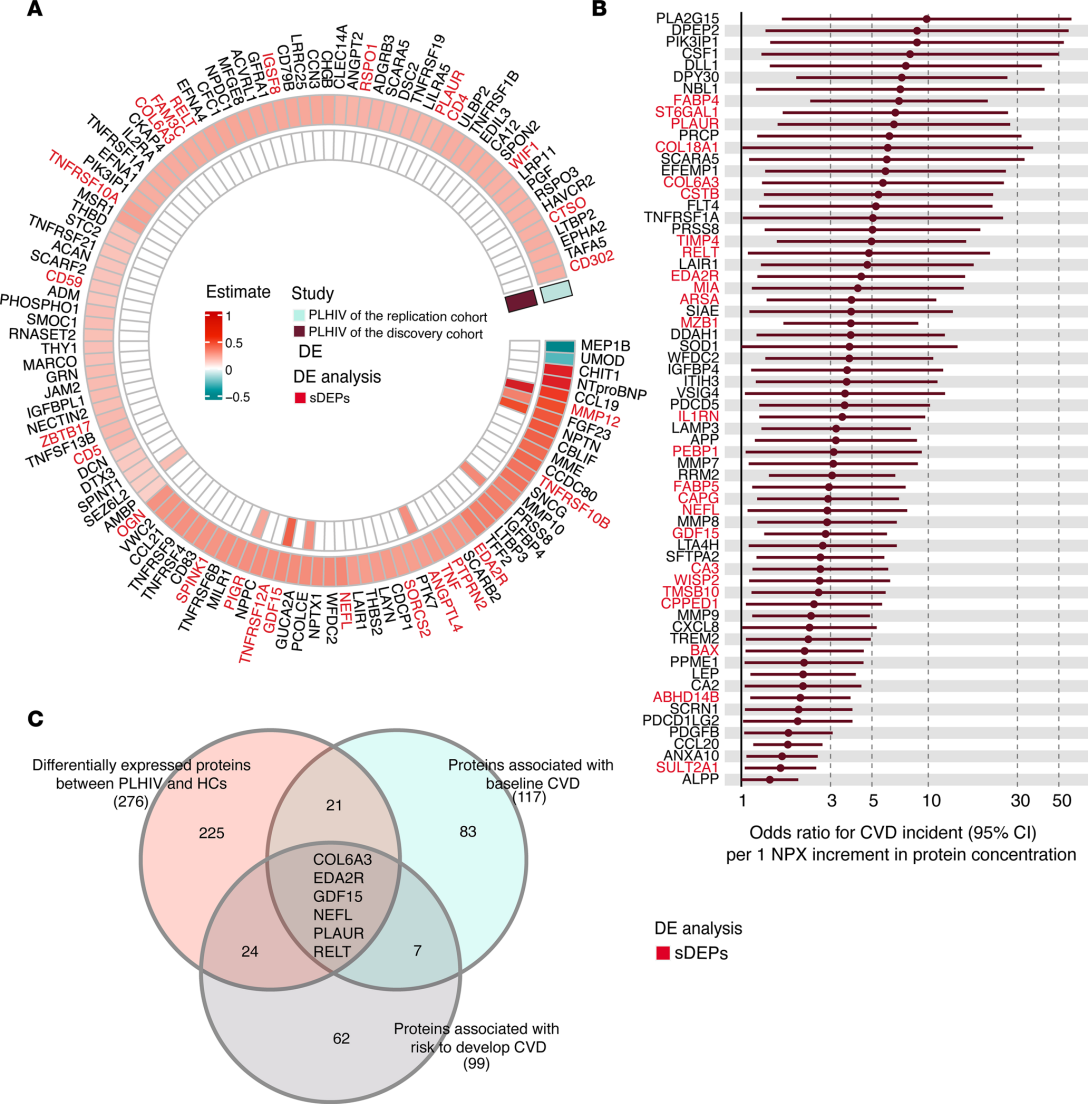
Plasma proteomic signatures of CVD in PLHIV. (**A**) Circular heatmap of associations of plasma proteins and CVD in PLHIV from the discovery (*n* = 205) and replication (*n* = 639) cohorts. sDEPs are highlighted in red. The associations were explored using linear regression corrected for age, sex, BMI, and smoking status. Only significant associations in the discovery (*P* < 0.05) and/or replication cohort (FDR < 0.05) are shown. (**B**) Forest plot showing positive associations between proteins and CVD events in PLHIV in the discovery cohort (*P* < 0.05; *n* = 205). *P* values were calculated using a binomial logistic regression model adjusted for age, sex, BMI, smoking status, and the presence of dyslipidemia, hypertension, and type 2 diabetes at baseline. The relative effects of protein concentration are presented as ORs with 95% CIs. See also Supplemental Table 10. (**C**) Venn diagram showing the number of shared proteins identified in 3 groups: (i) sDEPs, (ii) proteins associated with the presence of CVD at baseline, and (iii) risk of developing CVD events during 5-year follow-up.

To investigate CVD-associated proteomic signatures in PLHIV, we first examined the associations of approximately 1,500 proteins with the presence of CVD at baseline in 2 independent HIV cohorts. We identified 117 proteins associated with CVD in 639 PLHIV (FDR < 0.05), of which 9 associations could be replicated in 205 PLHIV (*P* < 0.05). Among the 117 proteins associated with CVD, 27 (23%) were found to be upregulated in PLHIV (Figure 4A). The identified associations include the well-known cardiac injury biomarker NT pro-BNP. Furthermore, we examined whether plasma proteins were associated with the risk of developing CVD in PLHIV. During the 5-year follow-up, 10% of PLHIV in the discovery cohort experienced a cardiovascular event (Table 1 and Supplemental Table 10). After correcting for demographic and well-known risk factors for CVD, such as age, sex, BMI, smoking status, and the presence of dyslipidemia, hypertension, and type 2 diabetes at baseline, 99 proteins were identified as associated with the probability of experiencing a cardiovascular event (*P* < 0.05, binomial logistic regression), among which 29 (30%) were known to be upregulated in PLHIV (Figure 4B and Supplemental Table 11). Of note, sDEPs were significantly enriched for association with the risk of developing CVD in PLHIV (*P* = 0.021) (Supplemental Figure 7). In contrast, only IL-6 from the established markers of the inflammation panel showed significant associations with the risk of developing CVD (Supplemental Table 12). Upregulated proteins in PLHIV that had the highest ORs include those that are involved in lipid metabolism (FABP4 [OR, 6.9; *P* = 0.0004]; FABP5 [OR, 2.9; *P* = 0.02]; ARSA [OR, 3.8; *P* = 0.01]), sialylation (ST6GAL1 [OR, 6.6; *P* = 0.007]), immune response (PLAUR [OR, 6.5; *P* = 0.01], RELT [OR, 4.8; *P* = 0.3], IL1RN [OR, 3.4; *P* = 0.01], GDF15 [OR, 2.8; *P* = 0.007], MZB1 [OR, 3.8; *P* = 0.001]), extracellular matrix organization and/or degradation (COL18A1 [OR, 6; *P* = 0.04], COL6A3 [OR, 5.7; *P* = 0.02], MIA [OR, 4.2; *P* = 0.03], TIMP4 [OR, 4.9; *P* = 0.007]), regulation of proteolysis (CSTB [OR, 5.4; *P* = 0.01], PEBP1 [OR, 3.1; *P* = 0.03]), inducing apoptosis (EDA2R [OR, 4.3; *P* = 0.02]), actin binding (CAPG [OR, 2.9; *P* = 0.01]), and axon development (NEFL [OR, 2.8; *P* = 0.03]).

Next, we explored protein overlap among the following groups: (i) DEPs in PLHIV compared with HC, (ii) proteins associated with CVD at baseline, and (iii) proteins associated with the risk of developing CVD. We identified 6 upregulated proteins in PLHIV — GDF15, NEFL, PLAUR, RELT, COL6A3, and EDA2R — that were simultaneously associated with the presence and risk of developing CVD (Figure 4C and Supplemental Figure 8). Moreover, we explored the potential causal relationship of these proteins with CVD using publicly available results from Mendelian randomization for 1,002 plasma proteins on 225 phenotypes through the PheWAS database (36). GDF15, RELT, and PLAUR were associated with more than 2 CVD-related traits (Supplemental Table 13); COL6A3, EDA2R, and NEFL were not included in the database.

## Discussion

In this study, high-throughput proteomic profiling was performed to assess the DEPs between PLHIV and HCs, using independent discovery and replication cohorts. Specifically, we profiled nearly 1,500 plasma proteins that mirror inflammatory, oncologic, neurologic, and cardiovascular processes using a proximity extension assay coupled with the next-generation sequencing technology. Furthermore, we explored the cell and tissue origin of sDEPs as well as the pathways they are involved in. Furthermore, we investigated the relationship of gut microbial species with the sDEPs, especially those originating from intestinal tissues. Finally, we investigated the plasma proteomic signature of CVD in PLHIV.

Compared with HCs, 276 plasma proteins involved in inflammatory, cardiometabolic, oncologic, and neurologic processes were differentially expressed in PLHIV, suggesting a systemic dysregulation of circulating protein concentrations in PLHIV treated long term with ART. Most of the identified DEPs were upregulated (*n* = 266 of 276; 96%), which was a likely novel finding for 236 of the 276 proteins (86%), with some being identified in previous proteomic studies (13–17, 19, 20, 22), as depicted in the Supplemental Table 4.

Using publicly available transcriptomic data from different tissues, most of the sDEPs were found to be derived from the intestine and lymphoid tissues, which substantiates the presence of gut perturbations and the attributed persistent gut immunity dysregulation (37), even in patients with chronic, well-treated HIV. Indeed, the intestine harbors the largest mass of lymphoid tissue in the body (gut-associated lymphoid tissue), which is an essential component of local and systemic immune defense. On the single-cell level, the sDEPs seem to be expressed from proximal enterocytes, intestinal goblet cells, and a variety of innate (dendritic and Langerhans cells) and adaptive immune cells (T and plasma cells), suggesting disturbances of nutrient absorption, gut mucosal barrier, and immunity. Association between the proportions of sDEPs and immune cells was verified using flow cytometry, which showed a substantial relation between sDEPs and the proportions of various adaptive and innate immune cells, particularly a decrease in the proportion of the Th17 to Treg ratio. Furthermore, sDEPs were strongly enriched in the chemokine signaling pathway and other innate immune response–related pathways, including neutrophil degranulation, chemokine signaling, and NK cell–mediated cytotoxicity. Activation of adaptive immunity was also represented by antigen processing and presentation and MHC class II antigen presentation pathways, as well as lipid metabolism pathways including PPAR signaling pathway, highlighting sustained perturbation of immune regulation and lipid metabolism despite adequate treatment in PLHIV. Intestine- and lymphoid tissue–specific proteins were enriched in the innate immune response and/or lipid metabolism–related pathways, pointing toward a possible interplay between gut innate immunity and intestinal lipid absorption that relates to persistent intestinal damage and inflammation.

Although most of the gut mucosa studies primarily assessed CD4^+^ T cell disturbances in ART-naive individuals (38), a previous study reported changes in gut innate immune cell distributions, such as neutrophil infiltration in colorectal biopsy specimens of PLHIV receiving ART (39). Because of limitations in studying the disruption of gut immunity in PLHIV, most of the findings on HIV-induced immune gut damage were derived from macaque studies (38). In line with our findings, studies using a pathogenic, SIV-infected, nonhuman primate (NHP) model showed disturbances of several gut mucosal cytokines and chemokines, including CXCL8, CXCL9, CXCL10, CCL25, and TNF (38, 40–42). In contrast to the progressive and pathogenic SIV infection NHP model that includes the Indian and Chinese subtypes, natural SIV infection in African NHPs is asymptomatic and characterized by a low level of immune activation (38, 43, 44). Furthermore, African NHPs also showed lack of increase in hypercoagulability and cardiovascular pathology despite highly replicating virus (45). Of note, a study has shown the potential role of gut damage in relation to HIV disease progression, with evidence suggesting that African NHPs maintain gut epithelial integrity and lack evidence of microbial translocation (46). Gut integrity may protect African NHPs from developing intestinal dysfunction and chronic inflammation, which are known to drive both HIV disease progression and associated comorbidities. These findings suggest that gut damage may be a key factor in the progression of HIV and warrant further investigation into potential therapeutic interventions targeting the gut barrier.

Interestingly, the sDEPs that showed the highest fold-change (≥1.5) in PLHIV were proteins that are highly expressed in the intestine, such as CES3, FBP1, and members of the FABP family, such as FABP1, IFABP, FABP6, and RBP2, as well as FABP5, which is expressed in the intestine to a lesser extent (26, 47). FABPs are known as cytoplasmic chaperones of long-chain fatty acids, eicosanoids, and other lipids and are linked to multiple metabolic and chronic inflammatory diseases (47–49). FABPs regulate a variety of cellular processes, such as inflammation, immunity, metabolism, and energy homeostasis. Upregulation of FABPs in PLHIV may indicate an increased energy demand and metabolism, given that immune activation is associated with energy production and biosynthesis.

Gut mucosal homeostasis is regulated by extensive crosstalk between innate immune cells and gut microbiome. Herein, we report finding gut bacterial interaction with a large number of proteins in the plasma of PLHIV. Specifically, we identified that increased relative concentration of sDEPs — IL32, FABP6, and CCL11, to name a few — was strongly associated with a higher abundance of a variety of enriched bacterial species in PLHIV (*Prevotella sp*. *CAG 1092, Prevotella sp*. *CAG 279,* and *Prevotella sp*. *CAG 520*). Of note, gut bacterial species were more likely to be enriched with intestine- and lymphoid tissue–specific sDEPs compared with other sDEPs not specific to the intestine and lymph nodes. Despite the modest sample size of our study, we were able to identify associations between protein concentrations and bacterial species that were previously reported using 92 CVD-related proteins in a healthy population (*n* = 1,264), including EPCAM, TFF3, and REG3A (36). Moreover, we observed associations between the intestinal and lymphoid tissue–specific proteins with higher level of microbial translocation (IFABP) and systemic inflammation markers (sCD163, sCD14, hsCRP, IL6, and D-dimer), respectively. Altogether, our findings substantiate the interplay among bacterial dysbiosis and translocation, gut barrier integrity, and systemic immune responses in PLHIV receiving ART.

During the highly active ART era, CVD has emerged as an important cause of morbidity among HIV-infected individuals. Six upregulated proteins in PLHIV (GDF15, NEFL, PLAUR, RELT, COL6A3, and EDA2R), compared with healthy individuals, were associated with both the presence and the risk of developing CVD, and none of the established markers of inflammation, except IL-6. Only 2 proteins, GDF15 and EDA2R, were associated with HIV clinical parameters. GDF15, a cytokine belonging to the TGF-β family, is widely expressed by various cell types and strongly produced in response to inflammation or tissue injury (50). In line with our findings, elevated GDF15 has been reported in PLHIV with cardiovascular dysfunction (50, 51). GDF15 was previously identified as a prognostic biomarker for disease progression and mortality in patients with CVD (53–56) and linked to CVD incidence in the healthy population (57, 58). Urokinase plasminogen activator receptor (PLAUR) is associated with inflammation and immune activation (59) and is suggested as a marker of metabolic disturbances in PLHIV (60). Increased plasma PLAUR levels have been associated with an increased risk of CVD in PLHIV (61) and the general population (62–65). Furthermore, NEFL is an intermediate filament protein abundantly expressed in neurons and is a marker of neuronal injury. NEFL has been reported to be elevated following ischemic cerebrovascular events (66, 67) and can predict stroke incidents in people with diabetes mellitus (68). A recent report on the Framingham Heart Study highlighted the prognostic value of NEFL for CVD incidence, including coronary heart disease and heart failure but not stroke (69). In the context of HIV, NEFL levels are associated with HIV-related neurodegenerative disorders (70–72). RELT is a member of the TNF receptor superfamily, and it is especially abundant in hematologic tissues (73). Reports on RELT in relation to CVD are scarce, with only one study reporting an association between RELT and CVD incident, particularly myocardial infarct, in an elderly male population (74). Collagen VI α3 chain (COL6A3) is one of the most abundantly expressed collagens in adipose tissue. Although we found no study that reported a relation with CVD, COL6A3 has been associated with CVD risk factors, such as obesity, inflammation, and insulin resistance (75–77). Finally, EDA2R is a member of the TNF receptor family that may act as an inducer of apoptosis (78, 79). However, we found no study that reported its relation with CVD. Although the biological mechanisms underlying the associations with CVD are still unclear, publicly available Mendelian randomization studies provided evidence of a causal association among GDF15, RELT, and PLAUR with CVD-related traits (Supplemental Table 13) (80). In addition, the therapeutic potential of GDF15 in the treatment of cardiometabolic disease is currently the subject of ongoing research (81). Together, our findings underscore the value of the proteins GDF15, NEFL, PLAUR, RELT, COL6A3, and EDA2R as potential targets for CVD prevention in PLHIV.

Some limitations of our study should be considered. First, the disparities in baseline demographics between groups may introduce bias in the study findings. Therefore, a regression model with baseline adjustment was applied in all analyses to correct for imbalance in baseline demographics, such as age, sex, and smoking status. Second, protein origin was predicted using publicly available transcriptomic data sets at baseline. Thus, gut mucosal single-cell transcriptomic studies in PLHIV treated long term with suppressive ART are needed to broaden our understanding of dysregulation of intestinal immunity in patients with chronic HIV. Next, being a cross-sectional study, further investigation through functional assays and/or Mendelian randomization studies in the context of HIV are needed to delineate the causal relationship of the identified associations. Nevertheless, our findings on 6 protein markers associated with CVD were based on a 2–time point assessment spanning a 5-year follow-up period. This supports that the described proteins are not only linked to baseline data but highlight their potential to predict the risk of comorbidities development in PLHIV. More studies with a larger sample size with an assessment of individual CVD events are needed to validate the clinical utility of these proteins on CVD risk assessment.

Using high-throughput proteomic profiling, a systemic dysregulation of protein concentration in PLHIV, compared with HCs, was identified, which was validated using 2 independent discovery and replication cohorts of PLHIV and HCs. The identified upregulated proteins pointed to possible dysregulation of cellular processes related to innate immune activation in intestinal layers and related immune tissues. Furthermore, the following upregulated proteins in PLHIV, namely, GDF15, NEFL, PLAUR, RELT, COL6A3, and EDA2R, may serve as potential biomarkers and novel targets for therapeutic approaches for CVD in PLHIV receiving ART.

## Methods

### Study cohorts

This study used data from 2 independent cohorts of PLHIV, the 200HIV and 2000HIV cohort, and HCs, from the 500 and 200 Functional Genomics (FG) cohorts, which are part of the HFGP (23).

#### Discovery cohort.

Proteomic profiling was generated from 211 PLHIV of the 200HIV cohort and 122 age- and sex-matched HCs from the 500FG (*n* = 56) and 200FG (*n* = 65) cohorts, hereafter referred to jointly as the discovery cohort. The 200HIV cohort was previously described (82, 83). Briefly, PLHIV from the 200HIV cohort were enrolled between December 2015 and February 2017 and consisted of PLHIV at least 18 years old who received ART for longer than 6 months and had HIV-RNA levels of fewer than 200 copies/mL. Exclusion criteria included HIV-2 infection, signs of acute intercurrent infections, use of antibiotics for less than 1 month prior to enrollment, pregnancy, or active hepatitis B or C infection. For all participants, baseline data on comorbidities and comedication were available (Table 1). Furthermore, PLHIV from this cohort were followed up for 5 years after study inclusion (up to 2021), and relevant clinical events were documented. The 500FG and 200FG studies comprised healthy individuals who were at least 18 years old and were enrolled between 2013–2014 and 2018, respectively.

#### Replication cohort.

An independent cohort of PLHIV and HCs used for replication studies, hereafter referred to jointly as the replication cohort. Proteomic profiling was performed using the first 646 participants of the 2000HIV cohort (ClinicalTrials.gov NCT03994835) (84) and 100 age- and sex-matched HCs from the 200FG cohort. PLHIV from the 2000HIV cohort were recruited from 4 HIV treatment centers (Radboud UMC Nijmegen, Erasmus MC Rotterdam, OLVG Amsterdam, Elisabeth-Tweesteden Ziekenhuis Tilburg). Baseline data on comorbidities and comedication were available for all the participants. The 2000HIV and 200FG participants were enrolled between 2019–2021 and 2019, respectively. Similar inclusion and exclusion criteria were applied as for the discovery cohort.

#### Proteomic profiling.

Collection, processing, and storage of the discovery and replication cohorts’ samples were performed according to study protocols from the HFGP (23). Venous whole-blood samples were collected using EDTA tubes and centrifuged into plasma before being stored at –80°C. Proteomic profiling was performed in plasma samples by Olink Proteomics AB using a proximity extension assay coupled with next-generation sequencing as a readout method (84). For this study, we used the Olink Explore 1536 platform, consisting of 1,472 proteins assigned into four 384-well multiplex panels focused on inflammation, cardiometabolic, oncologic, and neurologic proteins.

### Plasma inflammatory markers

Established inflammatory markers and an intestinal barrier dysfunction marker in PLHIV were measured in the plasma samples of the PLHIV in the discovery cohort (*n* = 211). Absolute plasma concentrations of acute-phase protein hsCRP, monocyte activation markers (sCD14 and sCD163), D-dimer, and IFABP were quantified using ELISA (R&D Systems for all ELISAs except D-dimer, which was from Abcam) according to the manufacturer’s protocols. IL-6 was measured using SimplePlex Cartridges (Protein Simple).

### Immunophenotyping and gating strategies

Immunophenotyping data of PLHIV in the discovery cohort were analyzed on the basis of a protocol and gating strategies that have been described previously (82). Blood samples were stained and measured on a 10-color Navios flow cytometer with solid-state lasers. Five supplemental, 10-color Ab panels were used for analysis. Flow cytometry data were processed using Kaluza software, versions 1.3 and 2.1. Gating was independently verified by 2 specialists. Absolute numbers of leukocyte (CD45^+^) cell subsets were calculated using the absolute number of WBCs per milliliter of blood, as measured by the Beckman Coulter AcT Diff Hematology Analyzer. A subset of 33 whole-blood cell populations, representing innate and adaptive cell compartments, were selected for analysis. Data were presented as the percentages of WBCs based on the cell count of each subpopulation relative to its respective population (1 level up).

### Metagenomic data generation and profiling

Metagenomics data were available for 152 PLHIV participants in the discovery cohort (86). Fecal DNA was isolated using the QIAamp Fast DNA Stool Mini Kit (QIAGEN; no. 51604) and sequenced by Novogene using the Illumina HiSeq platform whole-genome shotgun sequencing. Reads were processed using KneadData (version 0.7.4) to remove low-quality reads and contamination of the human genome by filtering reads that aligned to the human reference genome (version NCBI37). MetaPhlAn3 (version 3.0.7) was used to determine microbial taxonomic profiles by mapping reads to species-specific pan-genomes with UniRef90 annotations. Unclassified reads were translated and aligned to a protein database. Bacteria present in less than 20% of the samples were removed.

### Statistics

#### Quality control of proteomic data.

In each of the 4 panels from the Olink Explore 1536 platform, IL-6, CXCL8, and TNF were measured as technical duplicates for QC purposes. Strong correlations were found between technical duplicates (IL-6, CXCL8, and TNF; mean Spearman’s correlation coefficient *r* > 0.9) for both the discovery and replication cohorts. To exclude duplicated proteins, IL-6, CXCL8, and TNF measurements were selected from the inflammatory panel, resulting in 1,463 unique protein assays for downstream data analyses. Proteins were delivered as normalized protein expression (NPX) values, which is a relative protein quantification unit and reported on the log_2_ scale. QC per sample and normalization were performed in-house by Olink services. Specifically, samples with at least 500 mean assay counts and deviation within ±0.3 NPX for either incubation and amplification controls passed the QC (85). The average intra- and interplate CVs among all assays were not greater than either 15% and 30% for the discovery and replication cohorts, respectively.

For protein differential expression (DE) analysis, QC per protein was performed to remove proteins with (i) NPX values below the limit of detection (LOD) in greater than 25% of the samples in both groups (PLHIV and HCs) and (ii) a difference in the LOD values between PLHIV and HCs of less than 20%. For protein association analysis within PLHIV, we excluded proteins with LOD values in greater than 25% of the samples. To detect outliers, we performed QC per sample using PCA. Samples falling above or below 3 SDs from the mean of principal components 1 (PC1) and/or 2 (PC2) were considered outliers and excluded. The overview of proteomic QC processes is depicted in Supplemental Figure 1.

#### Protein DE analysis.

We compared the relative concentration of plasma proteins between PLHIV and HCs of the discovery cohort and validated the findings in an independent replication cohort. In the discovery cohort, 7 outliers (*n* = 5 PLHIV and 2 HCs) were identified at sample-level QC and excluded from downstream data analyses, resulting in 205 PLHIV from the 200HIV and 120 HCs from the 500FG (*n* = 56) and 200FG (*n* = 64) cohort. After protein-level QC, 1,309 proteins were available in PLHIV and HCs participants in the discovery cohort (Supplemental Figure 1). In the replication cohorts, similar QC approaches were performed as in the discovery cohort. We included 1,337 proteins that passed protein-level QC as described above. In addition, 4 participants who did not use ART (elite controllers) and 4 additional outliers based on PCA analysis (*n* = 3 PLHIV and 1 HC) were excluded, resulting in 639 PLHIV and 99 HCs for DE analysis in the replication phase (Supplemental Figure 1). A list of included and excluded proteins and the abbreviations is presented in Supplemental Table 1. To visualize the extent to which the proteomic profile of PLHIV overlaps with HCs, we performed PCA analysis of 205 PLHIV and 120 HCs in the discovery cohort and 639 PLHIV and 99 HCs from the validation cohort (Figure 1B).

For DE analysis, we used a linear regression model with age, sex, and smoking status as covariates using the Limma R package. DE analysis was performed for 205 PLHIV and 102 HCs in the discovery cohort and 639 PLHIV and 84 HCs from the validation cohort for whom we have complete information on age, sex, and smoking status (Figure 1C). NPX values of protein levels were used for DE analysis in both the discovery and replication phase. Limma uses an empirical Bayes method to moderate the standard errors of the estimated log_2_ fold-changes (86). *P* values were adjusted for multiple testing comparisons using an FDR method, and proteins with FDR < 0.05 and *P* < 0.05 were considered statistically significant in the discovery and replication phases, respectively.

#### Tissue- and cell-specific protein enrichment analysis.

To calculate the enrichment of tissue- and cell-specific proteins in our DEPs, we applied a hypergeometric test using the enrichment function from bc3net R package using bulk and single-cell transcriptomic data from the HPA project (26, 87). HPA uses consensus transcriptomic data from HPA and GTEx (27) databases from 54 human tissues and 76 cell types to classify genes according to their tissue and single-cell type–specific origin.

The hypergeometric *P* value is calculated as:

 (Equation 1)
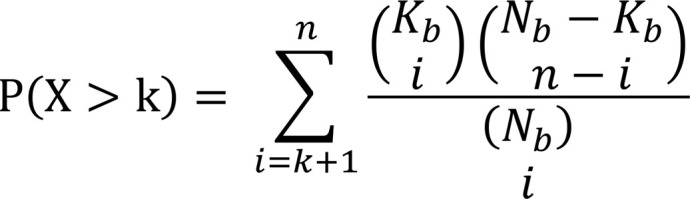


where *k* is the number of tissue- or single cell–specific genes in the candidate gene list, *n* is the number of proteins in the candidate protein list (*n* = X; referring to upregulated DEPs), *N_b_* is the total number of the reference/background proteins (*n* = 1,463), and *K_b_* is the total number of tissue- or cell-specific genes for a tissue or cell in the reference/background genes. The *P* values were corrected for multiple-hypothesis testing using the FDR method. We used tissues or cells with greater than 3 tissue- or cell-specific genes in the reference gene list for robust enrichment analysis. A significant *P* value (*P* < 0.05) means more proteins were detected in a particular tissue or cell type than expected.

#### Functional pathway enrichment analysis.

We performed a functional pathway enrichment analysis of DEPs using the Database for Annotation, Visualization and Integrated Discovery bioinformatics tool (88). The Kyoto Encyclopedia of Genes and Genomes and Reactome databases were used as a reference library, using a reference gene list of the genes that encode for the 1,463 unique proteins measured using the Olink explore panel. Pathways were considered significant with a *P* value of less than 0.05 and a protein count greater than 3. The pathway enrichment results were presented as a network using Enrichment Map (89). Nodes represent pathways and weighted edges represent the degree of gene overlap score between 2 pathways. The overlap score was calculated by the average between the jacquard and overlap coefficients. To identify clusters of highly overlapped pathways, nodes were connected if their overlap score was greater than 0.375. The R package AutoAnnotate was used to identify and annotate clusters of nodes based on the Markov cluster algorithm weighted algorithm (90).

#### Association between plasma proteins and immune cell proportions in PLHIV.

Prior to association analysis, immune cell proportion data were log_2_-transformed to follow a normal distribution. An association between plasma proteins and immune cell proportions was determined using a linear regression model adjusted for age, sex, and smoking status in 205 PLHIV in the discovery cohort.

#### Association between plasma proteins and gut bacterial composition in PLHIV.

Identification of bacterial compositions and differentially abundant bacterial species between PLHIV and HCs was previously performed in PLHIV in the discovery cohort (35). In short, differential abundance analysis of bacterial species between PLHIV and HCs (Dutch Microbiome Project study) identified 57 and 19 bacterial species that were enriched and depleted in PLHIV, respectively. Prior to analysis, relative abundances of the bacterial species were transformed using a centered log-ratio, and bacteria detected in less than 20% of participants were excluded. An association between plasma proteins and the relative abundance of bacterial species was determined using a linear regression model adjusted for age, sex, smoking status, and read counts in 143 PLHIV in the discovery cohort.

#### Association between plasma proteins and CVD in PLHIV.

We determined the association between the relative concentration of plasma proteins and the baseline incidence of CVD in 2 HIV cohorts (*n* = 120 and 639 PLHIV from the discovery and validation cohort, respectively) using a linear regression model adjusted for age, sex, BMI, and smoking status. CVDs referred to the presence of myocardial infarction, stroke, peripheral arterial diseases, and/or angina pectoris. Next, we assessed the prognostic value of protein relative concentration for the association with the risk of developing CVD during a 5-year follow-up in a group of PLHIV in the discovery cohort (*n* = 205). For this, we used a binomial logistic regression model adjusted for age, sex, BMI, smoking status, and the presence of relevant comorbidities, including dyslipidemia, hypertension, and type 2 diabetes. The causal relationship between proteins and CVD was further assessed using publicly available results from protein Mendelian randomization analysis in the PheWAS database (80).

#### Data visualization.

All analyses and data visualization were conducted in R, version 4.0.2, unless otherwise stated. The following R packages were used for data visualization: ggbiplot for PCA plots; ggplot2 for pie charts, 4-quadrant plots, and bar charts; ComplexHeatmap for heatmaps; vcd for mosaic plots; circlize for circular plots; and ggforestplot for forest plots. The study overview was created using BioRender.

### Study approval

Ethical approval was granted by the Ethical Committee of the Radboud University Medical Center Nijmegen, the Netherlands (approval no. NL42561.091.12 [200HIV and 500FG]; 2018-399 EC [200FG]; and NL68056.091.81 [2000HIV]). Written informed consent was obtained from all participants involved in this study, and experiments were conducted according to the Declaration of Helsinki principles.

## Author contributions

AJAMVDV, MGN, LABJ, and QDM contributed to the conceptualization, study design, and data interpretation and led the project; WAJWV, ALG, MJTB, LEVE, MJC, LVDW, and JCDS contributed to participant recruitment, data collection, and laboratory analyses; NV, AJAMVDV, and VM contributed to the analysis design and interpretation; NV contributed to the formal analysis and data integration; YZ contributed to the microbiome analysis; NV, VM, and AJAMVDV wrote the original draft of the manuscript; and YZ, WAJWV, ALG, MJTB, LEVE, MJC, LVDW, JCDS, MHG, JF, QDM, LABJ, and MGN contributed to writing and editing the manuscript.

## Supplementary Material

Supplemental data

Trial reporting checklists

ICMJE disclosure forms

Supplemental tables 1-13

## Figures and Tables

**Table 1:**
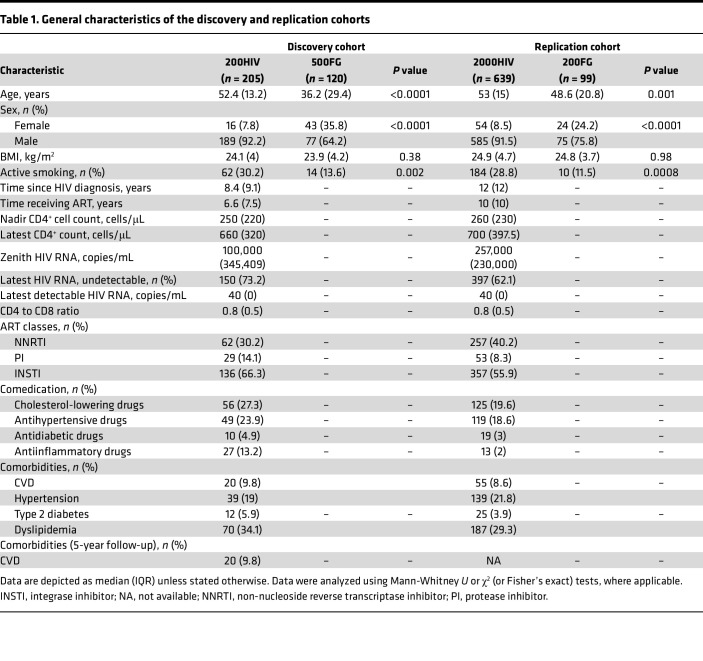
General characteristics of the discovery and replication cohorts

## Version 1 04/20/2023

In-Press Preview

## Version 2 06/08/2023

Electronic publication
