# Supplementary material for: High-throughput proteomic analysis reveals systemic dysregulation in virally suppressed people living with HIV
Source: JCI Insight. 2023 Jun 8;8(11):e166166. doi: 10.1172/jci.insight.166166 (PMC10393229; doi:10.1172/jci.insight.166166)
Supplement: Supplemental data [file jciinsight-8-166166-s208.pdf]

High-throughput proteomic analysis reveals systemic dysregulation in virally suppressed people living with HIV

Nadira Vadaq[1,2], Yue Zhang[3], Wilhelm A.J.W. Vos[1,4], Albert L. Groenendijk[1,5], Martinus J.T. Blaauw[1,6], Louise E. van Eekeren[1], Maartje Jacobs-Cleophas[1], Lisa van de Wijer[1], Jéssica Cristina dos Santos[1], Muhammad Hussein Gasem[2], Leo A.B Joosten[1,7], Mihai G. Netea[1,8], Quirijn de Mast[1], Jingyuan Fu[3,9], André J.A.M. van der Ven[1], Vasiliki Matzaraki[1]

1. Department of Internal Medicine, Radboudumc Center for Infectious Diseases, Radboud Institute of Health Science (RIHS), Radboud university medical center, Nijmegen, The Netherlands
2. Center for Tropical and Infectious Diseases (CENTRID), Faculty of Medicine, Diponegoro University, Dr. Kariadi Hospital, Semarang, Indonesia
3. Department of Genetics, University of Groningen, University Medical Center Groningen, Groningen, The Netherlands
4. Department of Internal Medicine, OLVG, Amsterdam, The Netherlands
5. Department of Medical Microbiology and infectious diseases, Erasmus Medical Center, Rotterdam, The Netherlands
6. Department of Internal Medicine, Elizabeth-Tweesteden Ziekenhuis, Tilburg, The Netherlands
7. Department of Medical Genetics, Iuliu Hatieganu University of Medicine and Pharmacy, Cluj-Napoca, Romania
8. Department of Immunology and Metabolism, Life and Medical Sciences Institute, University of Bonn, Bonn, Germany
9. Department of Pediatrics, University of Groningen, University Medical Center Groningen, Groningen, The Netherlands

Corresponding author:

Nadira Vadaq, Department of Internal Medicine, Radboud University Medical Center, Geert Grooteplein Zuid 10, 6525 GA Nijmegen, The Netherlands; e-mail: N.Nadira@radboudumc.nl

Conflict-of-interest statement:

ViiV Healthcare supported the 2000HIV Human Functional Genomics Partnership Program[1]

[1] ViiV Healthcare had no control on data quality, statistical analyses or final interpretation of the data.

Supplemental Figures

Supplemental Figure 1

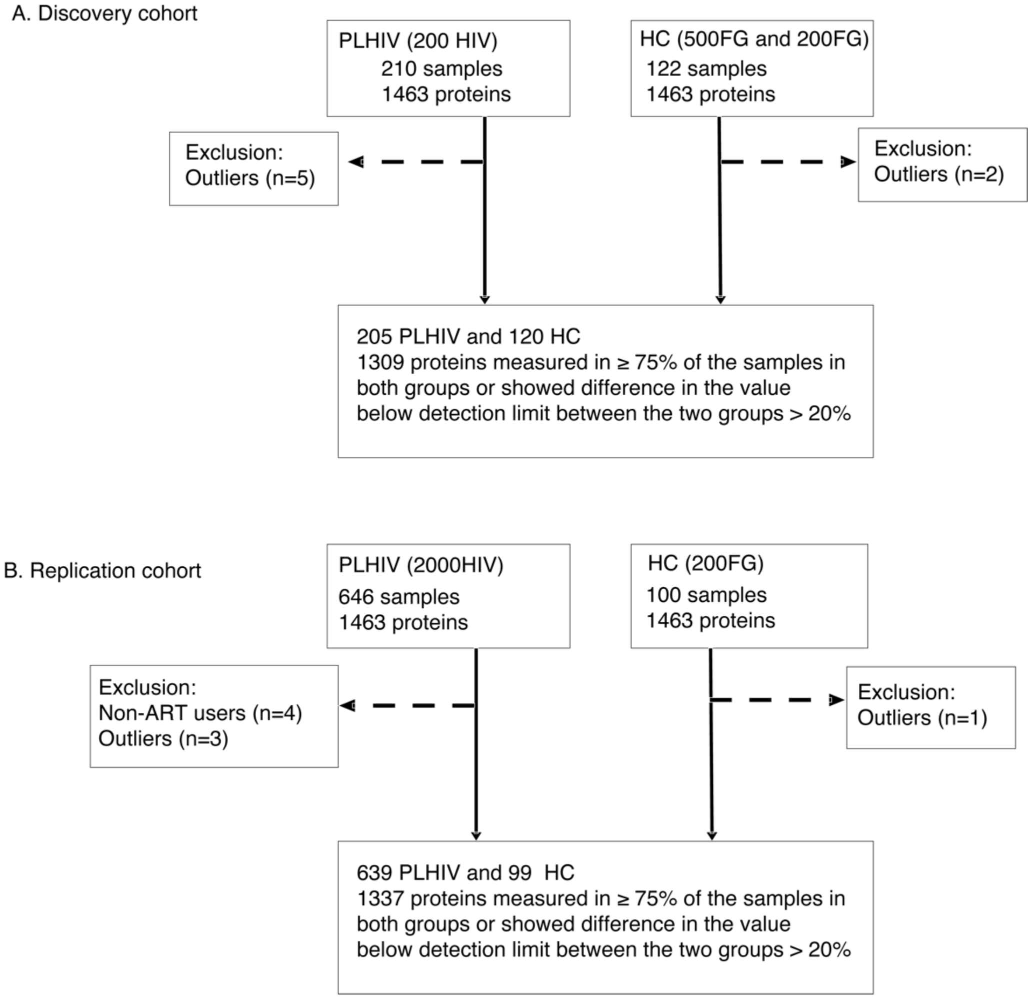

Supplemental Figure 1. Quality control of proteomic data

Schematic representation showing quality control (QC) per sample and per protein prior to downstream data analysis in the **(A)** discovery and **(B)** replication cohort. Outliers detection were done using principal component analysis (PCA), in which data points falling above and below three standard deviations (SD) from the mean of principal component one (PC1) and two (PC2) were excluded. Upon QC per protein, we used proteins that were (1) measured in at least 75% of samples from PLHIV or HCs, or (2) showed a difference in the value below the limit of detection (<LOD) between PLHIV vs HCs >20% (see Methods).

Supplemental Figure 2

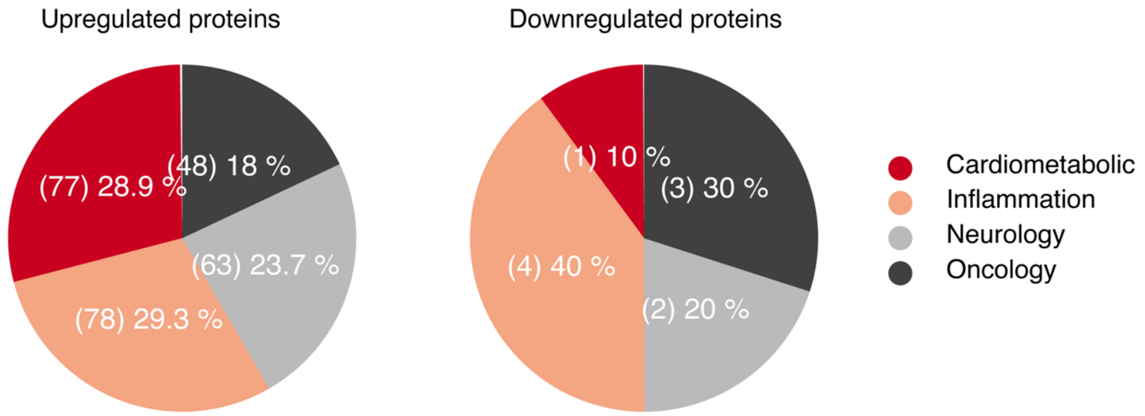

Supplemental Figure 2. Distribution of sDEPs across the cardiometabolic, inflammation, neurology and oncology panels.

Pie charts indicating the percentage of upregulated and downregulated differentially expressed proteins in PLHIV compared to HCs that were replicated using two independent cohorts (FDR <0.05; n=276 proteins). DE analysis was performed using a linear regression model with age, sex, and smoking status as covariates. Proteins were grouped based on their respective Olink panel.

Supplemental Figure 3

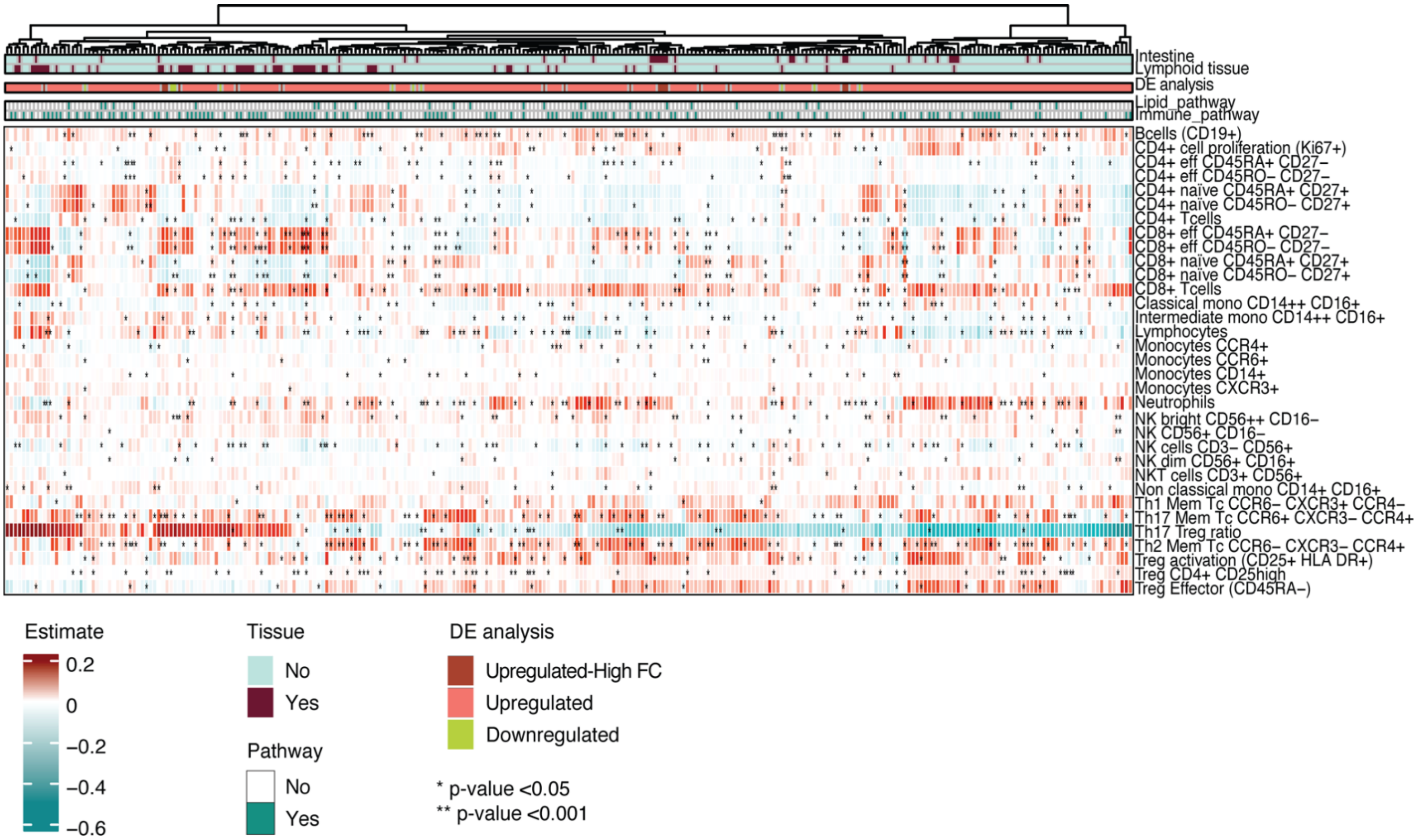

Supplemental Figure 3. sDEPs were associated with multiple innate and adaptive immune cells.

Heatmap showing the correlations between sDEPs (n = 276) and immunophenotyping data measured in PLHIV of the discovery cohort (n=205). The color-coding key depicts the beta estimate calculated by a linear regression model with age, sex, and smoking status as covariates. See also Supplemental Table 7.

Supplemental Figure 4

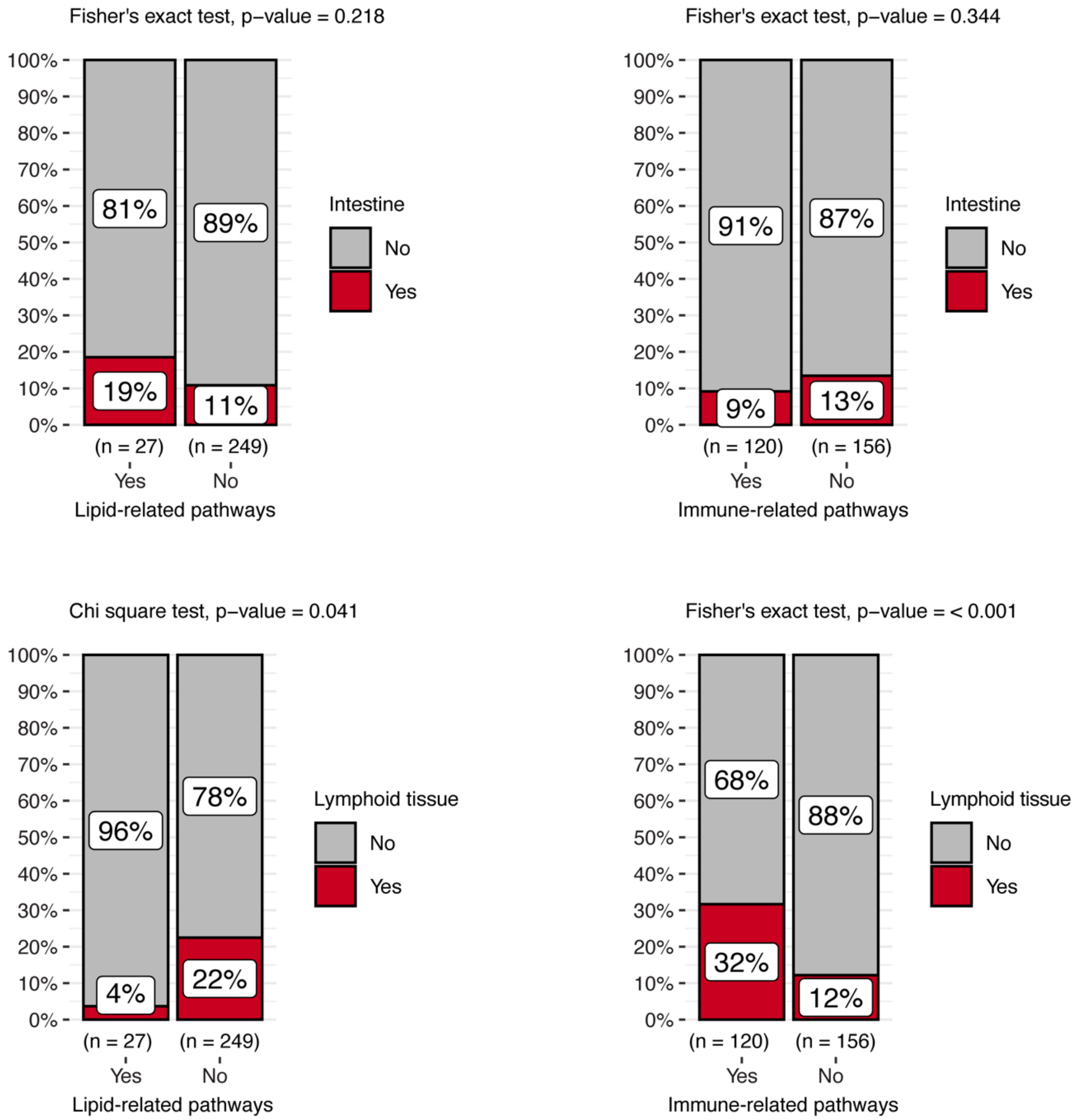

Supplemental Figure 4. Enrichment of sDEPs specific to the intestine/lymphoid tissues in the immune/lipid-related pathways.

Bar plots showing the percentage of sDEPs specific to the intestine/lymphoid tissues that were involved in the immune/lipid-related pathways. Enrichment p-values were obtained from the chi-square/Fischer exact test of independence.

Supplemental Figure 5

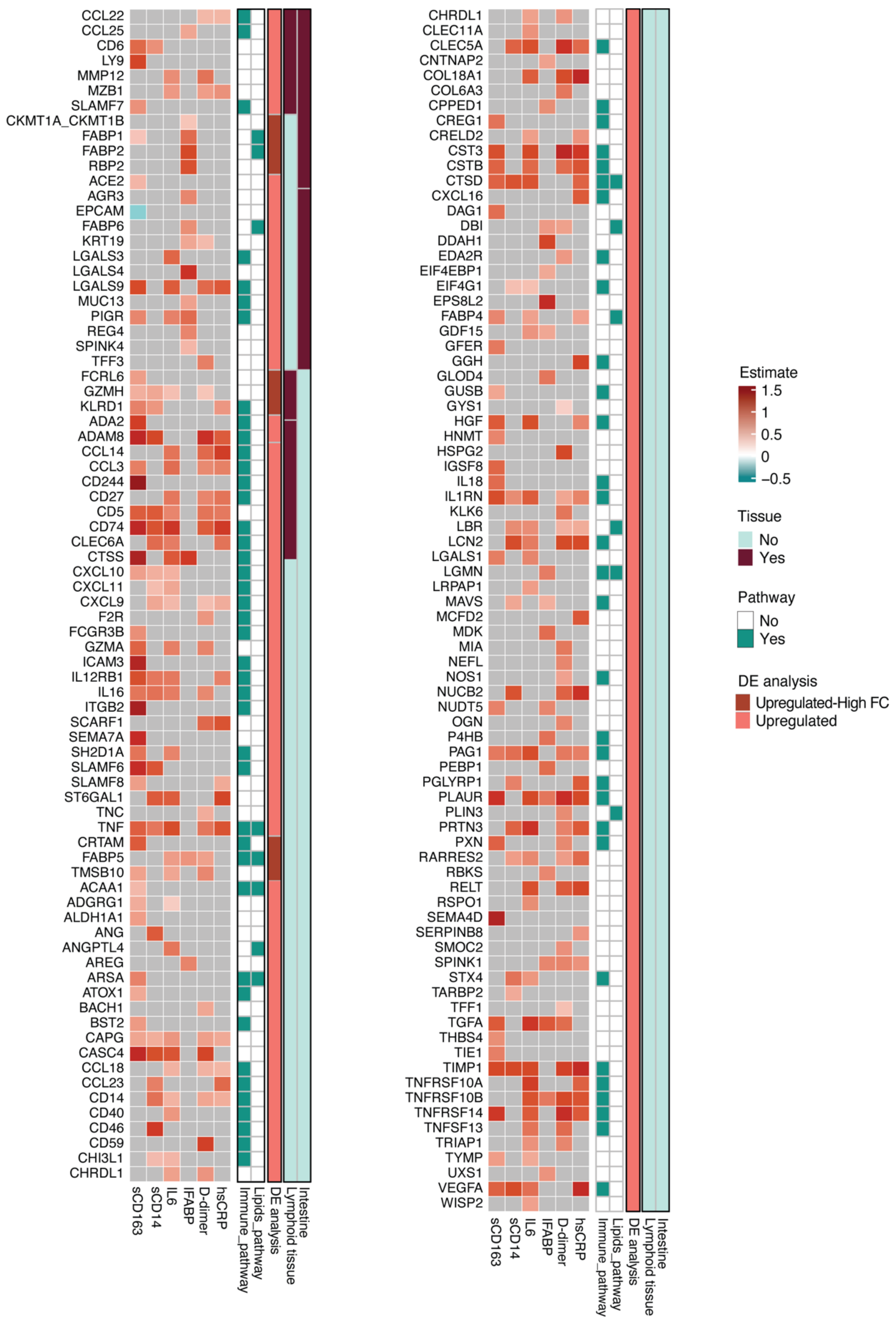

Supplemental Figure 5. sDEPs were strongly associated with established markers of systemic inflammation and microbial translocation.

Heatmap presenting associations between the sDEPs and absolute concentration of established markers of systemic inflammations (hsCRP, D-Dimer, IL6, sCD14, and sCD163) and microbial translocation (IFABP) in PLHIV from the discovery cohorts (n=205). The analysis was performed by linear regression adjusted for age, sex, and smoking status. Only significant associations (FDR <0.05) were shown.

Supplemental Figure 6

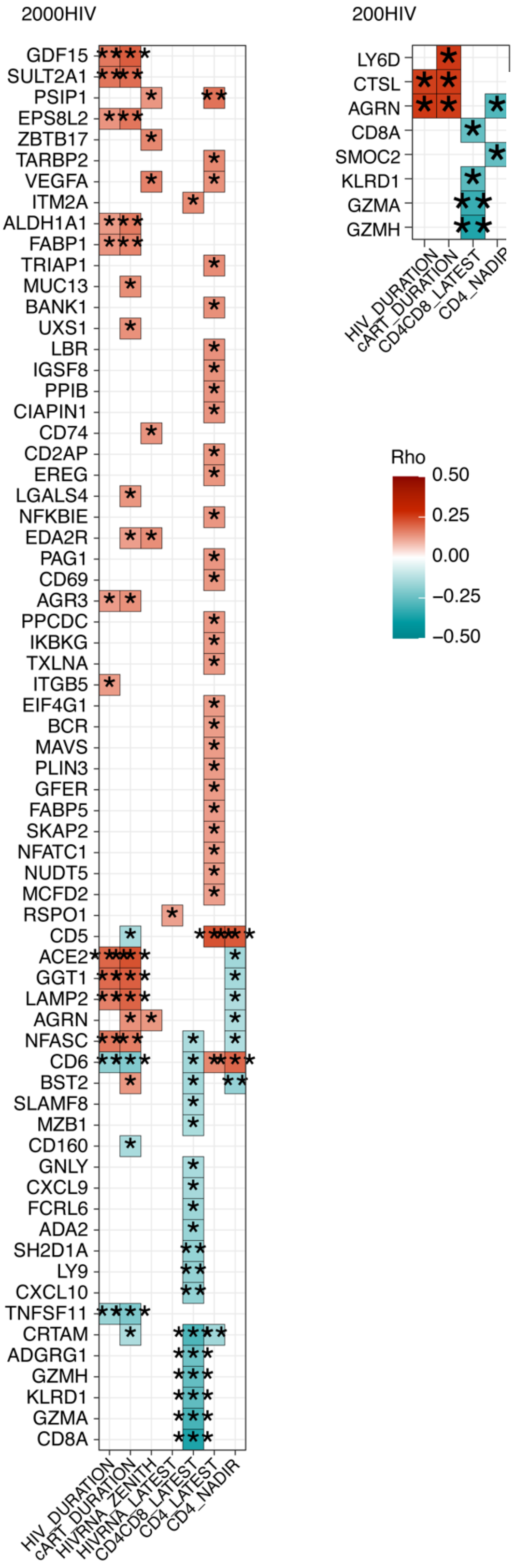

Supplemental Figure 6. Minimal associations between sDEPs and HIV-related clinical parameters

Heatmap presenting associations between the sDEPs and HIV-related parameters in PLHIV from the discovery (n=205) and replication cohorts (n=639). The analysis was performed by partial spearman correlation corrected for age, sex, and smoking status. Only significant associations (FDR <0.05) were shown. Significance level (FDR corrected) was defined as follows: <0.05(*), <0.005(**), and <0.0001(***).

Supplemental Figure 7

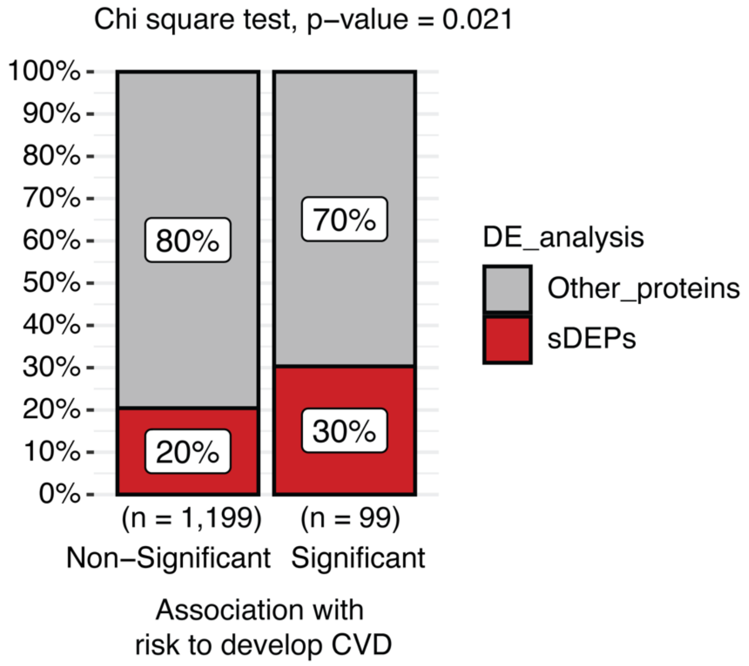

Supplemental Figure 7. sDEPs were enriched in the proteins associated with the risk of developing CVD.

Bar plots showing the percentage of sDEPs in the proteins associated with the risk of developing CVD. Analyses were performed using a binomial logistic regression model using age, sex, BMI, smoking status, dyslipidemia, hypertension, and type 2 diabetes as covariates in the PLHIV from the discovery cohort (n=205). The significance level was defined as p-value <0.05. Enrichment p-values were obtained from the chi-square test of independence.

Supplemental Figure 8

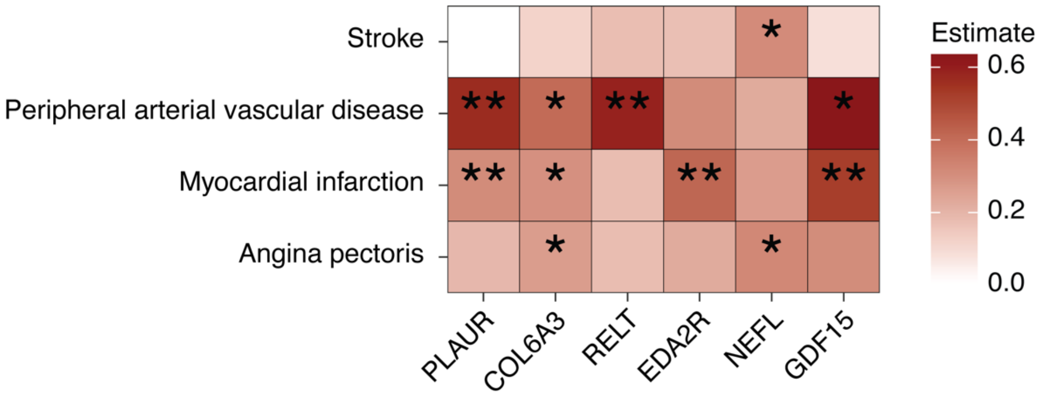

Supplemental Figure 8. Associations between PLAUR, COL6A3, RELT, EDA2R, NEFL, and GDF15 with the presence of CVD

Heatmap presenting associations between PLAUR, COL6A3, RELT, EDA2R, NEFL, and GDF15 with the presence of CVD in PLHIV from the replication cohort (n=639). The analysis was performed by linear regression model using age, sex, and smoking status as covariates. Significance level (FDR corrected) was defined as follows: <0.05(*), <0.005(**), and <0.0001(***).
